# Genome Sequence of Chiqui Virus, a Novel Reovirus Isolated from Mosquitoes Collected in Colombia

**DOI:** 10.1128/MRA.00881-18

**Published:** 2018-09-27

**Authors:** María Angélica Contreras-Gutiérrez, Hilda Guzman, Jedson F. Cardoso, Vsevolod L. Popov, Marcio R. T. Nunes, Sandra Uribe, Steven G. Widen, Thomas G. Wood, Nikos Vasilakis, Robert B. Tesh

**Affiliations:** a Grupo de Investigación en Sistemática Molecular, Universidad Nacional de Colombia, Medellín, Colombia; b Programa de Estudio y Control de Enfermedades Tropicales, Universidad de Antioquia, Medellín, Colombia; c Department of Pathology, Center for Biodefense and Emerging Infectious Diseases, University of Texas Medical Branch, Galveston, Texas, USA; d Center for Technological Innovation, Evandro Chagas Institute, Ministry of Health, Ananindeua, Pará, Brazil; e Postgraduate Program in Virology (PPGV), Evandro Chagas Institute, Ministry of Health, Ananindeua, Pará, Brazil; f Department of Biochemistry and Molecular Biology, University of Texas Medical Branch, Galveston, Texas, USA; g Center for Tropical Diseases and Institute for Human Infections and Immunity, University of Texas Medical Branch, Galveston, Texas, USA; University of Arizona

## Abstract

We report here the complete genome sequence of a novel reovirus, designated Chiqui virus (CHQV) strain CoB38d, that was isolated from a pool of unidentified mosquitoes collected in northern Colombia in 2013. CHQV has nine double-stranded DNA (dsRNA) genome segments and has similarity to viruses belonging to the family *Reoviridae*, subfamily *Spinareovirinae*.

## ANNOUNCEMENT

Viruses in the family *Reoviridae* are extremely diverse and infect a wide range of plants and animals. Members of this family are currently divided into 15 recognized genera, which are grouped into 2 subfamilies, *Sedorevirinae* and *Spinareovirinae*, based on their core virion structures (1, 2; see https://talk.ictvonline.org/taxonomy/p/taxonomy_releases#fragment-12227_taxonomy_release_history_panel_release_info). Here, we report the near-full-length genome sequence of a novel reovirus isolated from a pool of mosquitoes collected during arbovirus surveillance studies in the municipality of San Bernardo del Viento, Córdoba Department, Colombia, in 2013.

A pool of 50 unidentified mosquitoes, designated CoB38d, was triturated in diluent, as described previously (3). After centrifugation, the supernatant fluid from the insect homogenate was inoculated onto single flask cultures of mosquito (C6/36) and Vero 76 cells that were incubated at 28°C and 37°C, respectively (3). Cultures were examined daily for evidence of viral cytopathic effect (CPE). Moderate CPE was observed in the C6/36 culture on the fifth day of incubation; no CPE was seen in the Vero cell culture after 14 days. Supernatant fluid from the C6/36 culture was removed, and the cell monolayer was fixed and subsequently examined by transmission electron microscopy (3). In ultrathin sections of infected C6/36 cells, multiple double-shelled spherical particles of 45 to 55 nm in diameter were observed in the cytoplasm.

Nucleic acids were extracted from the C6/36 cell culture supernatant and subjected to high-throughput sequencing, using parallel sequencing on the HiSeq 1500 platform and an Illumina TruSeq RNA version 2 kit, following the manufacturer’s protocol. Trimmomatic version 0.22 was used to remove adapter sequences and low-quality base calls with the following parameters: LEADING, 35; TRAILING, 35; SLIDINGWINDOW, 5:35; and MINLEN, 35. *De novo* assembly of the reads was performed with ABySS version 1.9.0 with default parameters and k-mer lengths of 19 to 41. Reads were mapped to the assembled viral contigs using Bowtie 2 version 2.2.5, with local parameter settings. Of 16,635,368 read pairs of raw data, 10,232,149 pairs passed the filtering process, and 945,072 pairs aligned to the viral genome. The average read coverage depth across the genome was 2,960×, and the average read length was 46.6 bases. Genome annotation (using the InterProScan program) and identity calculations were performed using the Geneious package version 9.1.8 (4). Amino acid sequences were individually aligned using the PROMALS3D webserver (5). Phylogeny was inferred by maximum likelihood (ML) and Bayesian analyses implemented in RAxML version 8.2.11 (6) and MrBayes version 3.2.6 (7), respectively. Bootstrap support of 1,000 replicates (ML) and 2 million iterations (Bayesian) with 10% burn-in was applied.

The genomes of two distinct viruses were detected in the C6/36 culture of mosquito pool CoB38d. One was an orthomyxovirus, designated Sinu virus, which was described previously (3). The second, a reovirus designated Chiqui virus (CHQV), is described in this report.

CHQV consists of 25,028 bp. The sizes of segments 1 to 9 are 4,009 bp, 4,013 bp, 3,758 bp, 3,455 bp, 2,863 bp, 1,975 bp, 2,534 bp, 1,275 bp, and 1,146 bp, respectively. All the genome segments have only one open reading frame (ORF) encoding corresponding primary polyproteins.

The 9 double-stranded DNA (dsRNA) segments of CHQV share conserved terminal sequences that are distinct from those of other known species of the family *Reoviridae*. Based on percent identity and phylogenetic analyses (ML and Bayesian), CHQV is most closely related to Spissistilus festinus reovirus (SpFRV) and Acinopterus angulatus reovirus (AcARV), two reoviruses isolated from plant-feeding insects (alfalfa hopper and leaf hopper, respectively) (8). Results of the phylogenetic analysis and the low identities with other known reoviruses for the encoded gene products (RNA-dependent RNA polymerases [RdRps]) indicate that the CHQV sequences represent a novel virus within the subfamily *Spinareovirinae*. The name Chiqui virus (CHQV) is proposed; its genus is uncertain. Chiqui is a small village in the municipality of San Bernardo del Viento, near the site where the mosquitoes were collected.

### Data availability.

The genome sequences of Chiqui virus were submitted to GenBank with the accession numbers MG958619 to MG958627, which correspond to genomic segments 1 to 9, respectively.
